# Rapid automated antifungal susceptibility testing system for yeasts based on growth characteristics

**DOI:** 10.3389/fcimb.2023.1153544

**Published:** 2023-05-02

**Authors:** Jinhan Yu, Chun He, Tong Wang, Ge Zhang, Jin Li, Jingjia Zhang, Wei Kang, Yingchun Xu, Ying Zhao

**Affiliations:** ^1^ Department of Clinical Laboratory, State Key Laboratory of Complex Severe and Rare Diseases, Peking Union Medical College Hospital, Chinese Academy of Medical Science and Peking Union Medical College, Beijing, China; ^2^ Graduate School, Chinese Academy of Medical Sciences and Peking Union Medical College, Beijing, China; ^3^ Beijing Key Laboratory for Mechanisms Research and Precision Diagnosis of Invasive Fungal Diseases, Beijing, China; ^4^ Department of Clinical Laboratory, Peking University School and Hospital of Stomatology, Beijing, China

**Keywords:** antifungal susceptibility testing, growth characteristics, discrepancy, minimum inhibitory concentration, epidemiological cutoff value, clinical breakpoints, Droplet 48, Sensititre YeastOne

## Abstract

Fungal pathogens are a major threat to public health, as they are becoming increasingly common and resistant to treatment, with only four classes of antifungal medicines currently available and few candidates in the clinical development pipeline. Most fungal pathogens lack rapid and sensitive diagnostic techniques, and those that exist are not widely available or affordable. In this study, we introduce a novel automated antifungal susceptibility testing system, Droplet 48, which detects the fluorescence of microdilution wells in real time and fits growth characteristics using fluorescence intensity over time. We concluded that all reportable ranges of Droplet 48 were appropriate for clinical fungal isolates in China. Reproducibility within ±2 two-fold dilutions was 100%. Considering the Sensititre YeastOne Colorimetric Broth method as a comparator method, eight antifungal agents (fluconazole, itraconazole, voriconazole, caspofungin, micafungin, anidulafungin, amphotericin B, and 5-flucytosine) showed an essential agreement of >90%, except for posaconazole (86.62%). Category agreement of four antifungal agents (fluconazole, caspofungin, micafungin, and anidulafungin) was >90%, except for voriconazole (87.93% agreement). Two *Candida albicans* isolates and anidulafungin showed a major discrepancy (MD) (2.60%), and no other MD or very MD agents were found. Therefore, Droplet 48 can be considered as an optional method that is more automated and can obtain results and interpretations faster than previous methods. However, the optimization of the detection performance of posaconazole and voriconazole and promotion of Droplet 48 in clinical microbiology laboratories still require further research involving more clinical isolates in the future.

## 1 Introduction

In recent years, an increasing number of patients have developed risk factors for invasive fungal infection, and resistant fungal pathogens have become more widespread, particularly in medical centers that attend to patients with complex underlying diseases, such as immunocompromised patients, patients exposed to long courses of broad-spectrum antibiotics, and patients with implanted medical devices (Bassetti et al., 2020; Fisher et al., 2022). Key interventions to combat the spread and emergence of antifungal resistance include rapid detection and quantification of resistance, as well as antimicrobial stewardship (van Belkum et al., 2020). Antifungal susceptibility testing (AFST) provides minimal inhibitory concentration (MIC) of an antifungal agent to support clinicians in managing fungal infections, thereby tracking the emergence and spread of resistance and allowing comparison of agent activities (Berkow et al., 2020).

For over 30 years, only a few AFST methods have been developed and widely implemented (Berkow et al., 2020; van Belkum et al., 2020). There are several AFST methods for the detection of MICs for fungal isolates, including the broth microdilution (BMD) reference methods by the Clinical and Laboratory Standards Institute (CLSI) (Clinical and Laboratory Standards Institute, 2022a) and Antifungal Subcommittee of the European Committee on Antimicrobial Susceptibility Testing (EUCAST) (http://www.eucast.org/ast_of_fungi/). The BMD methods, as gold standards for AFST, are labor-intensive during microdilution plate preparation and have expensive antifungal powders; therefore, it is impractical for routine use in clinical microbiology laboratories (Berkow et al., 2020).

Commercial systems are more standardized, practical, and easy to use in clinical microbiology laboratories, and their interpretation is less subjective than that of standard BMD (Delma et al., 2020). Currently, a few methodologies, such as the Sensititre YeastOne (SYO) colorimetric antifungal panel (Thermo Fisher Scientific, Waltham, MA [formerly TREK Diagnostic Systems]), Vitek 2 yeast susceptibility panel (bioMérieux, Hazelwood, MO, USA), and gradient diffusion strips (bioMérieux, Hazelwood, MO; Liofilchem, Waltham, MA), are widely used for AFST in clinical laboratories (Berkow et al., 2020; Delma et al., 2020). Nevertheless, these methods require many manual operations or a limited number of drugs to be tested (Berkow et al., 2020; Durand et al., 2021). Miscellaneous methods are constantly emerging to better meet clinical laboratory application scenarios. In-fiber antibiotic susceptibility testing is fast, highly sensitive, and compatible with the Food and Drug Administration (FDA)-approved workflow in clinical settings (Farid et al., 2022). A rapid ultrasensitive detector uses a high reflectance coefficient at high incidence angles when light travels from low- to high-refractive-index media. It can detect extremely low cell densities (optical density ≥5 × 10 ^7^) that correspond to approximately 20 bacterial cells, or a single fungal cell in the detection volume (Cansizoglu et al., 2019). The new detection methods listed above exhibit good theoretical feasibility.

Fluorescent dye-based detection technologies have been widely used for clinical detection. However, the cost of SYO is too high for clinical application, and it is necessary to purchase a supporting sample-loading device; otherwise, the operation is cumbersome.

In this study, we introduced a novel, rapid, automated AFST system for yeasts based on growth characteristics. This method also uses fluorescent dyes, such as SYO, and is more automated and cheaper than the existing methods. The automated system of antimicrobial susceptibility testing, Droplet 48 (D48), uses a unique fluorescence detection technology to feed back the growth of pathogens through continuous detection of changes in fluorescence intensity of the panel; it reports the results in real time. It also avoids the subjectivity of interpreting results by reading MICs manually, and is simple to apply in clinical microbiology laboratories.

**Figure 1:**
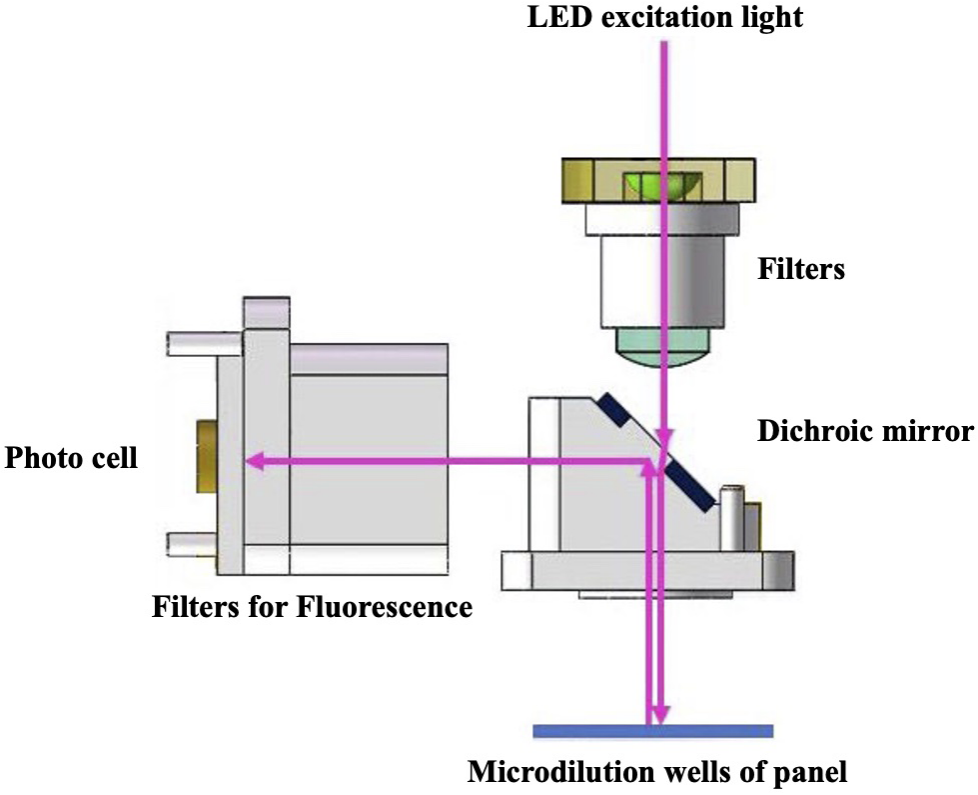
Automated antimicrobial susceptibility testing system, Droplet 48, uses the reflection fluorescence detection device to obtain fluorescence intensity change in microdilution wells. The fluorescence detection device has advantages of fast detection speed and avoidance of stray light interference. A stable light source with fixed band generated by monochromatic LED light source is used as an excitation light to illuminate the detection microdilution wells of the panel after filtering stray light using a filter and splitting by dichroic mirror. The panel continuously sampled the reflected light during the rotation process, and the fluorescence intensity was obtained through a high-sensitivity photocell. Software analysis processes the collected fluorescence data through a variety of algorithms to fit a characteristic growth curve.

**Figure 2:**
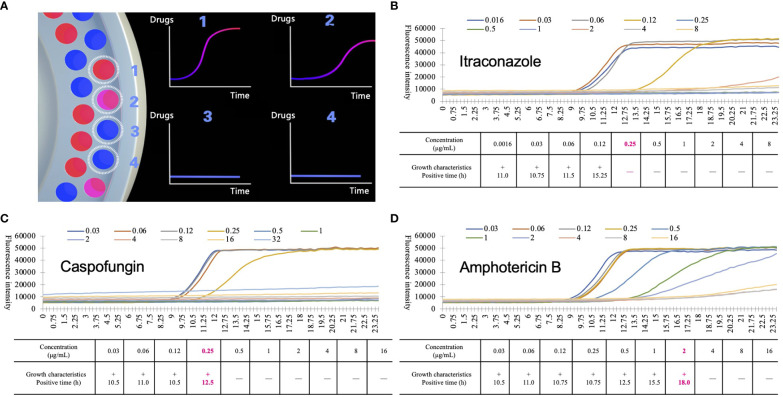
Detection principle of automated antimicrobial susceptibility testing system, Droplet 48 (D48). **(A)** the working mode diagram of D48: D48 monitors the fluorescence of microdilution wells containing different concentrations of antifungal agents in real-time and fits the growth characteristics of the strains by the fluorescence intensity over time. If the concentration of antifungal agents was higher than the minimum inhibitory concentration (MIC) of the tested strains (No.3 and No.4), the growth characteristics of the strains changed significantly compared to the microdilution wells containing lower MICs (No.1 and No.2, No.2 grows slightly slower than No.1); **(B-D)** growth characteristics of *Candida krusei* under different concentrations of itraconazole **(B)**, caspofungin **(C)** and amphotericin B **(D)**. During D48 testing, some substances will interfere with the fluorescence signal. Based on a large number of previous data, the MIC value of the agents were finally obtained by excluding the background signal and adding different correction algorithms. Boldfaced and red numbers indicate the MIC and positive reporting time of the antifungal agents.

The primary modes of propagation of pathogenic fungi are fission and proliferation, and their overall growth is exponential with a characteristic growth curve. Resazurin (blue) is a water-soluble dye that can be transformed into a fluorescent colorimetric indicator (pink) using metabolically active cells. When resazurin concentration was sufficient, the fluorescence intensity produced by resazurin was proportional to the number of viable cells (Zhang et al., 2017). Based on this principle, D48 uses resazurin as a redox indicator to detect fluorescence intensity and reflects the growth characteristics of pathogenic fungi in real time. Droplet 48 uses a reflective fluorescence detection device to obtain changes in fluorescence intensity in microdilution wells containing different concentrations of antifungal agents. Processed analysis software collected fluorescence data using various algorithms to fit a characteristic growth curve (**Figure 1**). Assuming that certain concentrations of antifungal agents can inhibit or kill microorganisms, the growth characteristics in this well will be significantly different from those of the control wells (antifungal agent-free), according to the threshold, slope, and acceleration algorithms (**Figure 2A**). The concentration of the antifungal agent in each well was considered the MIC. Considering *Candida krusei* as an example, the D48 detector fitted the acquired fluorescence signal to the growth curve and determined the MIC using various algorithms (**Figures 2B–D**).

**Figure 3:**
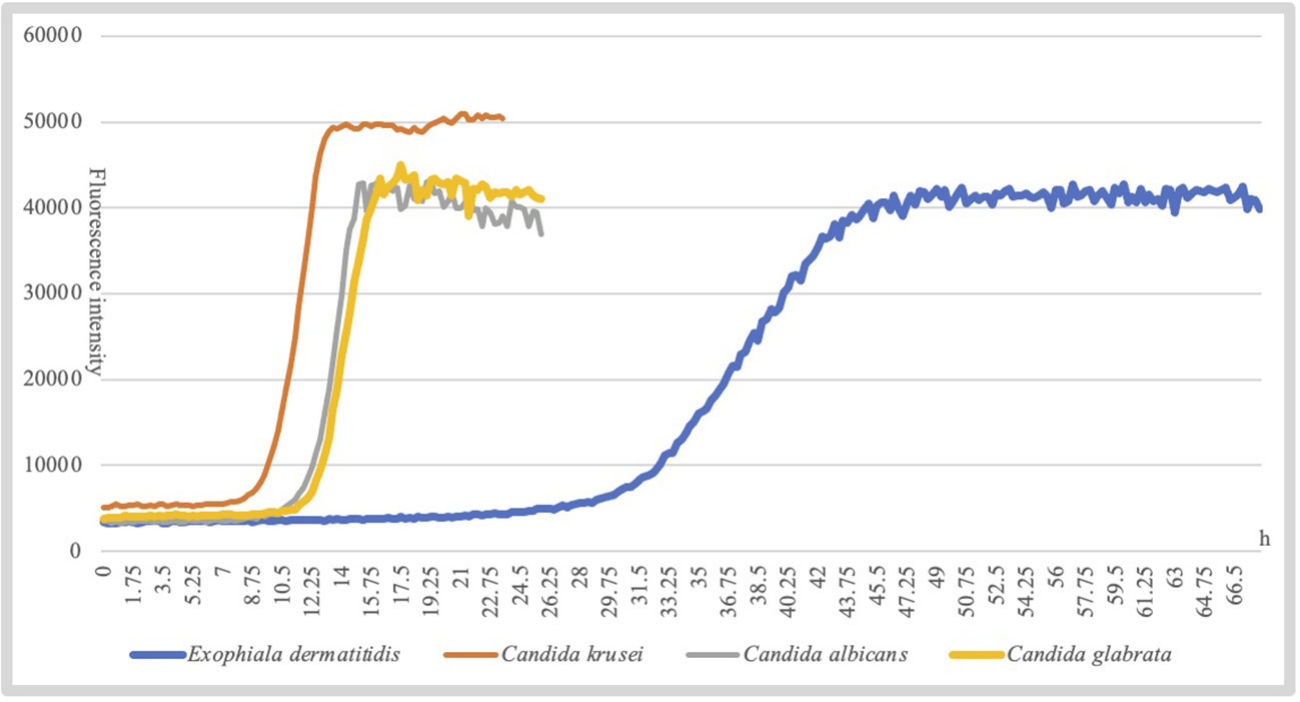
Different species of fungi slightly have different growth characteristics.

Additionally, different species of fungi exhibit slightly different growth characteristics under the same growth conditions. For example, *Candida albicans* entered the logarithmic growth phase at approximately 15 h, while *Exophiala dermatitidis* reaches it at approximately 45 h (**Figure 3**). To improve the accuracy of the assay, D48 combined the growth characteristics of the species to establish individualized parameters to interpret the threshold, slope, and acceleration values of the agent, thereby accurately interpret the antifungal susceptibility of the strain.

As required by the Clinical Laboratory Improvement Amendments U.D.o.H.a.H.S. (2023) each laboratory must verify whether it can obtain performance specifications comparable to those of the manufacturer (accuracy, precision [reproducibility], and reportable range of test results) before performing patient testing with a commercial AFST. Therefore, this study aimed to assess the concordance between the two methods for detecting AFST in commonly found clinical *Candida*, *Cryptococcus*, and some relatively rare fungi.

## 2 Materials and methods

### 2.1 Identification of strains and species

A total of 144 clinical fungal strains were randomly analyzed from the Peking Union Medical College Hospital and Peking University School and Hospital of Stomatology. Species of these strains were commonly found in patients with systemic infections, including 16 *Candida albicans*, 16 *Candida krusei*, 13 *Candida tropicalis*, 13 *Candida parapsilosis*, 13 *Candida lusitaniae*, 10 *Candida glabrata*, 10 *Candida guilliermondii*, and 10 *Cryptococcus neoformans*. All isolates were cultured from the blood or other sterile body sites (brain abscess and intra-abdominal samples). To fully validate the performance of D48, we included 10 strains of *Trichosporon asahii* isolated from patients with bloodstream infections, 22 strains of *Exophiala dermatitidis* causing oral mucosal infections, and 11 randomly selected isolates from patients with rare bloodstream infections.

The CHROMagar Candida chromogenic agar medium was used for initial identification. Species identification was further confirmed using matrix-assisted laser desorption ionization-time of flight mass spectrometry and sequencing of the nuclear ribosomal internal transcribed spacer region and large subunit of the 28S ribosomal DNA gene (D1/D2).

### 2.2 Antifungal susceptibility testing

**Figure 4:**
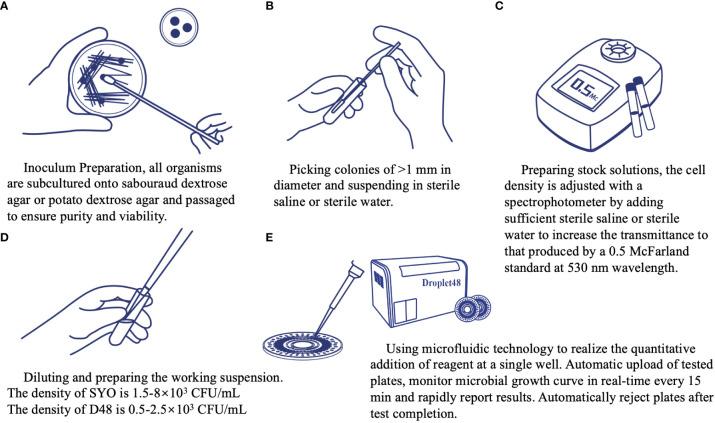
Operation procedure of the automated antimicrobial susceptibility testing system, Droplet 48.

The AFST with nine antifungal agents (fluconazole, voriconazole, itraconazole, posaconazole, caspofungin, micafungin, anidulafungin, amphotericin B, and 5-flucytosine) was performed, according to the manufacturer’s instructions. To prepare the inoculum, all the strains were subcultured onto sabouraud dextrose agar or potato dextrose agar at 35°C and subcultured again to ensure purity and viability (**Figure 4A**). Approximately five colonies of at least 1 mm in diameter were picked, suspended in sterile saline or water (**Figure 4B**), vortexed, and adjusted using a spectrophotometer to a transmittance equal to a 0.5 McFarland standard at a wavelength of 530 nm as a stock solution (**Figure 4C**). Further, 20 μL and 6 μL of stock solution were transferred to the SYO and D48 broth medium, and the final density of the working solution was 1.5–8 × 10^3^ CFU/mL and 0.5–2.5 × 10^3^ CFU/mL, respectively (**Figure 4D**). We ensured that the same stock solution tube was used in the experimental process of the two methods. Approximately 100 uL of stock solution was transferred into each microdilution well broth suspension to SYO panels and incubated without agitation at 35°C for 24 h before reading. An exception is *Cryptococcus* species isolates, which were maintained for 72 h before reading. Control wells were inspected for the presence and absence of growth. To get an accurate reading, plates with insufficient growth in the control well may be held for further 24 h. Between 3–3.2 mL of broth was transferred to the detection panel and added to the microdilution wells using the microfluidics technique. The instrument automatically completed the loading steps, quantitative sample addition, incubation, reporting of the results, and withdrawal (**Figure 4E**). To assess the reproducibility of the two methods, the AFST was repeated for isolates exhibiting MICs that differed by >1–2 two-fold dilutions. Quality control strains included *Candida parapsilosis* ATCC 22019 and *Candida krusei* ATCC 6258.

### 2.3 Data analysis

The MIC ranges, MIC_50_, and MIC_90_ were calculated using Microsoft Excel 2016 software. Clinical breakpoints (CBs) and epidemiology cut-off values (ECVs) in the 2022 CLSI files [M57S 4th Edition (Clinical and Laboratory Standards Institute, 2022b) and M27M44S 3rd Edition (Clinical and Laboratory Standards Institute, 2022a)] were used as the judging criteria for classification. When no CLSI CBs were used, specie-specific ECVs were used to define the isolates as wild type (WT) or non-WT. According to the CLSI guideline M52, essential agreement (EA) is the MIC obtained with D48 within two two-fold dilution steps from the MIC values detected by the SYO method. Categorical agreement (CA) was assessed and defined as the percentage of isolates classified in the same category (i.e., susceptible, intermediate, susceptible-dose-dependent, and resistant) by both methods. “Discrepancy” is used in a new AFST system when two systems are in disagreement, while “error” is used when the AFST result does not agree with the reference method result (such as BMD). Very major discrepancy (VMD) was defined as a test result when the D48 result is susceptible and SYO result is resistant. Major discrepancy (MD) was defined as a discrepancy in test results interpreted by D48 as resistant, and the comparator method result was susceptible. Minor discrepancy (mD) was intermediate, and the other was susceptible or resistant. The agreement between the D48 and SYO results was also assessed by calculating the Cohen’s kappa coefficient. The scale used to assess the degree of agreement was as follows: Kappa of ≤0.2, slight; 0.21–0.40, fair; 0.41–0.60, moderate; 0.61–0.80, substantial; and 0.81–1, almost perfect agreement (Wang et al., 2019).

This study was conducted in accordance with the Declaration of Helsinki and approved by the Ethics Committee of Peking Union Medical College Hospital (protocol code HS-3371, February 2022).

## 3 Results

The MICs for the quality control strains were within the recommended ranges of the CLSI for both assays. This study included 144 strains. Owing to inadequate growth, one *Saccharomyces cerevisiae* strain could not produce the desired results using the D48 method, and one *Rhodotorula mucilaginosa* strain could not obtain the desired results using the SYO method. Therefore, 142 samples were included in the statistical analyses.

The reportable range of D48 fully covered the CBs, ECVs, and quality control ranges of common clinically pathogenic fungi. Combined with microfluidics and other technologies to increase the concentration gradient of more drugs, D48 realizes simultaneous detection of 96 different drug concentrations. Except for 5-flucytosine and voriconazole, there was a slight difference in the reportable ranges between the two methods; however, the ranges were appropriate for clinical fungal isolates from China (**Table 1**). The reproducibility within ±2 two-fold dilutions was 100% for all the methods. An analysis of the agreement between D48 and SYO identification results is presented in **Table 1**. The EA (within two two-fold dilutions) between the two methods was best for fluconazole and micafungin (98.59%), followed by itraconazole (97.89%), voriconazole (97.18%), amphotericin B (95.07%), and caspofungin (92.96%). Posaconazole (86.62%) had the lowest EA, which was <90%. The CA of the four antifungal agents (fluconazole, caspofungin, micafungin, and anidulafungin) was >90%, except for voriconazole (87.93%). The highest CA value (98.72%) was observed for micafungin.

**Table 1:** Reportable range and geometric mean of antifungal agents used in this study and analysis on the agreement of D48 and SYO detection results.

Antifungal Agents	Reportable Range (μg/mL)		Geometric Mean (μg/mL)		%					Cohen’s kappa coefficient [95% confidence interval]
	D48	SYO	D48	SYO	EA [N_EA_/N_T_]	CA [N_CA_/N_CBs_]	VMD [N_VMD_/N_R_]	MD [N_MD_/N_S_]	mD [N_mD_/N_T_]	
Amphotericin B	0.03–16	0.12-8	0.76	0.54	95.07 [135/142]	—	—	—	—	0.47 [0.11–0.83]
Itraconazole	0.016–8	0.015-16	0.39	0.18	97.89 [139/142]	—	—	—	—	0.85 [0.57–1.14]
5-Flucytosine	0.06–64	0.06-64	0.09	0.31	92.25 [131/142]	—	—	—	—	—
Caspofungin	0.03–32	0.008-8	0.28	0.11	92.96 [132/142]	92.59 [73/78]	0	0	6.41 [5/78]	0.83 [0.70–0.97]
Fluconazole	0.25–256	0.12-256	1.84	2.41	98.59 [140/142]	92.30 [48/52]	0	0	7.69 [4/52]	0.89 [0.82–0.97]
Voriconazole	0.008–8	0.008-8	0.12	0.08	97.18 [138/142]	87.93 [51/58]	0	0	12.07 [7/58]	0.81 [0.70–0.92]
Anidulafungin	0.016–16	0.015-8	0.36	0.09	90.85 [129/142]	93.59 [73/78]	0	2.60 [2/77]	3.85 [3/78]	0.83 [0.69–0.97]
Micafungin	0.03-32	0.008-8	0.06	0.07	98.59 [140/142]	98.72 [77/78]	0	0	1.28 [1/78]	0.93 [0.85–1.00]
Posaconazole	0.016–2	0.008-8	0.15	0.12	86.62 [123/142]	—	—	—	—	0.06 [-0.16–0.27]

EA, essential agreement; minimal inhibitory concentration (MIC) result obtained with Droplet 48 (D48) that is within two two-fold dilution of the MIC value determined by the Sensititre YeastOne (SYO) method. N_EA_ is the number of tests that resulted in EA; CA, categorical agreement; agreement of susceptible, intermediate, susceptible-dose-dependent, and resistant results between the D48 and SYO methods. N_CA_ is the number of tests that result in CA, N_CBs_ is the number of tests that have the Clinical and Laboratory Standards Institute clinical breakpoints, very major discrepancy (VMD) is the major discrepancy, and VMD = (N_VMD_ × 100)/N_R_. N_VMD_ is the number of tests that result in a VMD, and N_R_ is the number of resistant microbial isolates as determined by SYO; MD, major discrepancy; MD = (N_MD_ • 100)/N_S_. N_MD_ is the number of tests that resulted in an MD; N_S_ is the number of susceptible microbial isolates as determined by SYO; mD, minor discrepancy, mD = (N_mD_ • 100)/N_T_, where N_mD_ is the number of tests that result in an mD, and N_T_ is the total number of isolates tested. If the MIC value of a method was identified as a range value (e.g., ≤2 or >4), the MIC value of another method that differed from its critical value within ±2 two-fold dilutions or within its range was also considered consistent (e.g., 8 vs. >2, 0.016 vs. ≤2, 2 vs. ≤4; 32 vs. >4); a horizontal thin line indicates no data available.

We also assessed the discrepancy between the SYO (comparator method) and D48 (**Table 1**). Two *Candida albicans* and anidulafungin showed MD (2.60%); no other MD or VMD agents were found. Voriconazole (12.07%), fluconazole (7.69%), caspofungin (6.41%), anidulafungin (3.85%), and micafungin (1.28%) were the agents with CBs that all showed mD. Categorical agreement analyses were performed for strains and agents with CBs only, and we used the Cohen’s kappa coefficient for further analysis of all the strains and agents with CBs or ECVs. We noticed an almost perfect degree of detection agreement between the D48 and SYO for fluconazole, voriconazole, itraconazole, caspofungin, micafungin, anidulafungin, and 5-flucytosine (Cohen’s kappa coefficient ≥0.81). Posaconazole (Cohen’s kappa coefficient: 0.06) showed slight agreement, whereas amphotericin B (Cohen’s kappa coefficient: 0.47) showed moderate agreement (**Table 1**).

In recent years, there has been growing interest in cases of uncommon and unknown “superfungal” diseases. In our study, 11 uncommon clinical fungal isolates from the bloodstream were included, and **Table 2** displays their MIC values. It is noteworthy that D48’s detection of *Trichosporon mucoides* and caspofungin/posaconazole, *Candida norvegensis* and anidulafungin, *Saccharomyces cerevisiae* and amphotericin B/fluconazole was more significant than two two-fold dilutions with SYO. The remaining samples (EA) were 100%. Although we could not interpret the consistency of the two detection methods due to the small number of strains, the MIC values of the tested strains were relatively consistent, which could provide a therapeutic option for patients with clinically rare fungal infections.

**Table 2:** Minimal inhibitory concentration (μg/mL) detection results of D48 and SYO for quality control strains and rare clinical infection fungi .

Isolates	Amphotericin B		Itraconazole		5-flucytosine		Caspofungin		Fluconazole		Voriconazole		Anidulafungin		Micafungin		Posaconazole	
	D48	SYO	D48	SYO	D48	SYO	D48	SYO	D48	SYO	D48	SYO	D48	SYO	D48	SYO	D48	SYO
ATCC 22019	0.5	0.5	0.12	0.12	0.25	0.5	0.5	0.25	2	2	0.06	0.03	0.5	0.5	0.5	1	0.06	0.06
ATCC 6258	2	1	0.5	0.25	8	8	0.5	0.25	16	32	0.25	0.25	0.12	0.06	0.25	0.25	0.25	0.25
*Saccharomyces cerevisiae*	**4**	**0.5**	0.25	0.12	≤0.06	≤0.06	0.25	0.12	**16**	**2**	0.06	0.03	0.25	0.12	*0.25*	*0.06*	0.12	0.25
	**16**	**0.5**	0.25	0.25	*0.25*	*≤0.06*	0.25	0.25	2	2	*0.25*	*.06*	0.12	0.12	0.25	0.12	*1*	*0.25*
	—	0.5	—	0.5	—	≤0.06	—	0.5	—	4	—	0.12	—	0.25	—	0.12	—	0.5
*Kodamaea ohmeri*	0.5	0.5	*0.5*	*0.12*	≤0.06	≤0.06	0.25	0.12	8	4	0.12	0.06	*0.5*	*0.12*	0.12	0.12	0.12	0.06
	0.5	0.5	0.25	0.12	≤0.06	≤0.06	0.25	0.25	8	16	0.12	0.06	0.25	0.25	0.06	0.12	0.12	0.06
*Pichia pastoris*	*4*	*1*	0.5	0.5	16	8	0.25	0.5	64	128	*0.25*	*1*	0.12	0.06	0.12	0.12	0.25	0.5
*Rhodotorula mucilaginosa*	1	—	4	—	≤0.06	—	0.12	—	≤0.25	—	4	—	>16	—	2	—	≤0.016	—
*Candida nivariensis*	2	1	0.25	0.5	*1*	*0.25*	0.12	0.06	4	4	0.06	0.12	*0.06*	*≤0.015*	≤0.03	0.015	*0.12*	*0.5*
*Candida norvegensis*	*1*	*0.25*	*0.5*	*0.12*	*16*	*4*	*0.12*	*0.03*	16	16	0.25	0.12	**0.12**	**≤0.015**	0.06	0.03	0.12	0.06
*Trichosporon mucoides*	1	0.5	0.25	0.25	16	32	**2**	**>8**	4	4	0.25	0.12	>16	>8	>32	>8	**1**	**0.12**
*Lodderomyces elongisporus*	0.5	0.25	0.06	0.12	1	0.5	0.12	0.06	0.5	0.5	0.016	≤0.008	*0.06*	*≤0.015*	≤0.03	0.03	0.06	0.12

“—” Indicates failure to detect minimal inhibitory concentration (MIC) of the isolate and antifungal agents; Italicized numbers indicate the discrepancy between the two antifungal susceptibility testing (AFST) methods was within ±2 two-fold dilutions for antifungal agents; Boldfaced numbers indicate the discrepancy was more than ±2 two-fold dilutions for antifungal agents. SYO, Sensititre YeastOne; D48, Droplet 48.

## 4 Discussion

A novel automated antimicrobial susceptibility testing system, D48 (Shanghai Fosun Long March Medical Science Co., Ltd.), based on the growth characteristics of pathogenic fungi, was evaluated in this study. It is real-time, quick, affordable, and simple to promote in clinical pathogenic microbiology laboratories, and avoids the subjectivity of manually reading MICs. The Fungi and Mycosis Research Center of Peking University compared the detection results of D48 and CLSI BMD methods when applied to the China Medical Device Product Registration Certificate (No.20192220368 and No.20192400349), and the EA of nine antifungal drugs were all >90%, which is consistent. Therefore, this study did not compare D48 with the BMD reference method.

According to the CLSI criteria for the verification of commercial antimicrobial susceptibility testing systems, SYO approved by the U.S. FDA was chosen as the comparator method. Importantly, the CLSI M52 document stipulates that when a clinical laboratory desires to implement a new AFST system, it should be compared with the commercial method that the laboratory is already using and meets the criteria.

The D48 should be verified for specific agent/fungus combinations tested when 1) CA and EA (if reporting MICs) are ≥90% compared to that in the current system; 2) VMD rate was <3% of total resistant isolates; and 3) MD rate was <3% for all susceptible isolates (Clinical and Laboratory Standards Institute, 2015). According to our study, all the agents and strains met the CLSI laboratory criteria, except for posaconazole (EA rate, 86.62%) and voriconazole (CA rate, 87.93%).

The Cohen’s kappa coefficient for amphotericin B was 0.47, while the EA rate was 95.07%. The Cohen’s kappa coefficient was calculated for strains with ECVs values, which reduced the sample data size compared with the EA included in all samples for calculation. Additionally, one *Candida albicans*, one *Cryptococcus neoformans*, and one *Trichosporon asahii* had inconsistent amphotericin B ECVs between the two methods, thereby leading to this discrepancy. Because the EA of the two methods was better than 90% for amphotericin B, we believe that the D48 has excellent performance in detecting amphotericin B.

The agreement between the two methods has also been evaluated in different species. The lowest CA rate (70%) was found for *Candida glabrata* and caspofungin, followed by 75% for *Candida albiacans* and voriconazole, 81.25% for *Candida albicans* and anidulafungin, 84.62% for *Candida parapsilosis* and fluconazole, and 87.50% for *Candida krusei* and voriconazole/caspofungin. The CA of the other strains and agents were >90%. Except for *Candida albicans* and anidulafungin whose MD was 12.5%, none of the isolates showed VMD or MD. *Candida glabrata* and caspofungin were associated with up to 30% mD, followed by *Candida albicans* and voriconazole (25.0%), *Candida parapsilosis* and fluconazole (15.38%), and *Candida krusei* and voriconazole (12.50%). The test range, MIC_50_, MIC_90_, and agreement rate between D48 and SYO for each species are shown in **Supplementary Table 1**. Since a sample size <30 is insufficient to identify discrepancies around the CBs while testing to verify the new antifungal susceptibility testing system, the agreement rate between some strains and antifungal agents was <90%, or MD was >3%, possibly because the number of tested strains was too small. The detection accuracy of different species should be verified by testing additional isolates.

**Figure 5:**
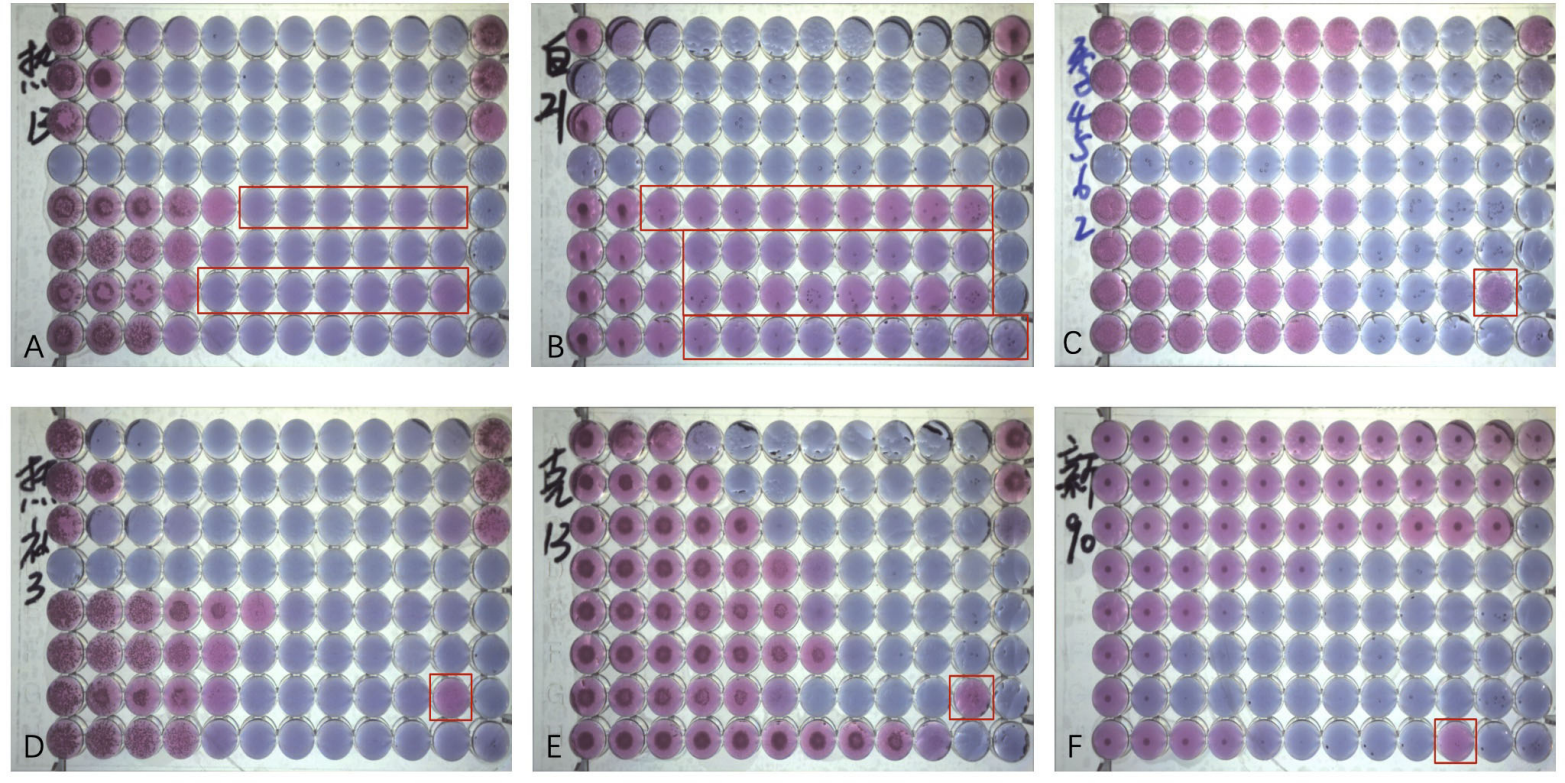
Antifungal Susceptibility Testing using Sensititre YeastOne (SYO) colorimetric antifungal panel (Thermo Fisher Scientific). **(A)** Weak “trailing growth” of posaconazole and itraconazole in *Candida tropicalis*. **(B)** Heavy “trailing growth” of posaconazole, voriconazole, itraconazole, and fluconazole in *Candida albicans*; **(C)** “Eagle effect” with itraconazole in *Candida guilliermondii*; **(D)** "Eagle effect" with itraconazole in Candida tropicalis. **(E)** “Eagle effect” with itraconazole in *Candida krusei*; and **(F)** “Eagle effect” with fluconazole in *Cryptococcus neoforman*.

In our study, the MIC was occasionally not determined accurately by the SYO because of “trailing growth” (Luna-Tapia et al., 2019). This phenomenon was mainly observed in *Candida albicans* and *Candida tropicalis*, which appeared as slight color changes and persisted in microdilution wells with concentrations greater than the MIC value (**Figures 5A, B**). According to several studies, *Candida tropicalis* has distinct phenotypes and genotypes for azole resistance and trailing (Astvad et al., 2018; Chen et al., 2021a; Chen et al., 2021b). Heavy trailing isolates were less susceptible to voriconazole, although weak trailing isolates (<25% of the positive growth control) were common and did not impair voriconazole efficacy (Astvad et al., 2018). Our research found that it is more challenging to precisely estimate how much “trailing growth” has occurred, and that this problem still has to be addressed in future development of the AFST technology.

Some strains can occasionally survive and grow in concentrations above the MIC, a phenomenon described as the “paradoxical growth effect” or “Eagle Effect” (Wagener and Loiko, 2017) of itraconazole and voriconazole (**Figures 5C-F**). It has been about 70 years since the “Eagle Effect” was first described for bacterial species. However, the “caspofungin paradoxical effect” (CPE) and other echinocandins have been frequently reported (Wagener and Loiko, 2017; Valero et al., 2020; Valero et al., 2022). However, its occurrence in azoles has not been reported. Because of the importance of the calcium/calcineurin/transcription factor-CrzA pathway in the regulation of CPE, one study on *Aspergillus fumigatus* discovered that 100% of ΔcrzAAf293 conidia did not exhibit CPE, while all ΔcrzACEA17 conidia did. A phenotype that should be regarded as antifungal tolerant is called CPE, a genetically encoded adaptive trait (Zhao et al., 2022). To determine whether the “paradoxical growth effect” can prevent invasive fungal diseases from being treated effectively in clinical settings, it is necessary to have a thorough understanding of the mechanisms underlying this phenomenon, as well as additional research on whether it exists in the human body.

In conclusion, we developed an innovative AFST system, D48, based on the growth characteristics of pathogenic fungi for the rapid determination of antifungal susceptibility profiles of fungal strains. Fluconazole, itraconazole, caspofungin, micafungin, anidulafungin, amphotericin B, and 5-flucytosine are seven regularly used clinical antifungal agents, and that D48’s sensitivity detection performance was highly consistent with that of SYO. Therefore, D48 can be considered an optional assay that is more automated than the currently widely used commercial methods, and can provide results and interpretation more quickly than the traditional methods. However, further research involving more clinical isolates is required to optimize the detection performance of posaconazole/voriconazole and establish the validity of the D48 assay.

## Data availability statement

The original contributions presented in the study are included in the article/**Supplementary Material**. Further inquiries can be directed to the corresponding author.

## Ethics statement

The study was conducted in accordance with the Declaration of Helsinki and approved by the Ethics Committee of Peking Union Medical College Hospital (protocol code HS-3371, February 2022).

## Author contributions

All authors listed have made a substantial, direct, and intellectual contribution to the work, and approved it for publication.
