# Supplementary material for: Rapid automated antifungal susceptibility testing system for yeasts based on growth characteristics
Source: Front Cell Infect Microbiol. 2023 May 2;13:1153544. doi: 10.3389/fcimb.2023.1153544 (PMC10185846; doi:10.3389/fcimb.2023.1153544)
Supplement: Supplementary file 1 [file Table_1.docx]

Supplementary Material

Rapid automated antifungal susceptibility testing system for yeasts based on growth characteristics

Jinhan Yu^1,2,3^, Chun He^4^, Tong Wang^1,3^, Ge Zhang^1,3^, Jin Li^1,3^, Jingjia Zhang^1,3^, Wei Kang^1,3^, Yingchun Xu^1,3*^, Ying, Zhao^1,3*^

^1^ Department of Clinical Laboratory, State Key Laboratory of Complex Severe and Rare Diseases, Peking Union Medical College Hospital, Chinese Academy of Medical Science and Peking Union Medical College, Beijing 100730, China

^2^ Graduate School, Chinese Academy of Medical Sciences and Peking Union Medical College, Beijing 100730, China

^3^ Beijing Key Laboratory for Mechanisms Research and Precision Diagnosis of Invasive Fungal Diseases, Beijing 100730, China

^4^ Department of Clinical Laboratory, Peking University School and Hospital of Stomatology, Beijing 100081, China

*** Correspondence:**

1. Ying Zhao (E-mail: zhaoying28062806@163.com; Telephone: +86-01069159788)

2.Yingchun Xu (E-mail: xycpumch@139.com; Telephone: +86-01069159766)

## Supplementary Table 1. Test MIC range, MIC_50_, MIC_90_ and agreement rate between the D48 and SYO methods by species. MIC, Minimal inhibitory concentration; SYO, Sensititre YeastOne; D48, Droplet 48

| Species (No. of isolates) and Antifungal Agents | D48 | | | SYO | | | % Of isolates | | | |
| --- | --- | --- | --- | --- | --- | --- | --- | --- | --- | --- |
| ***Candida albicans* (**16) | MIC range | MIC50 | MIC90 | MIC range | MIC50 | MIC90 | CA | VMD | MD | mD |
| Amphotericin B ^b^ | 0.25-4 | 0.5 | 1 | 0.5-1 | 0.5 | 1 | — | — | — | — |
| 5-flucytosine | ≤0.06-1 | 0.12 | 0.5 | ≤0.06-0.5 | 0.12 | 0.25 | — | — | — | — |
| Itraconazole | 0.03-0.5 | 0.12 | 0.25 | 0.03- | 0.12 | 0.25 | — | — | — | — |
| Voriconazole | ≤0.008-1 | 0.06 | 0.5 | ≤0.008-1 | 0.015 | 0.25 | 75.00 | 0 | 0 | 25.00 |
| Posaconazole ^b^ | ≤0.015-1 | 0.12 | 1 | 0.015-0.25 | 0.03 | 0.12 | — | — | — | — |
| Fluconazole | ≤0.25-1 | 0.5 | 4 | ≤0.12-4 | 0.5 | 4 | 93.75 | 0 | 0 | 6.25 |
| Anidulafungin | 0.03-2 | 0.12 | 1 | ≤0.015-0.12 | 0.03 | 0.12 | 81.25 | 0 | 12.50 | 6.25 |
| Micafungin | ≤0.03 | ≤0.03 | ≤0.03 | ≤0.008-0.015 | 0.015 | 0.015 | 100 | 0 | 0 | 0 |
| Caspofungin | 0.06-0.25 | 0.12 | 0.25 | 0.015-0.06 | 0.06 | 0.06 | 100 | 0 | 0 | 0 |
| ***Candida krusei*** (16) | MIC range | MIC50 | MIC90 | Test range | MIC50 | MIC90 | CA | VMD | MD | mD |
| Amphotericin B ^b^ | 0.5-4 | 1 | 2 | 0.25-1 | 0.5 | 1 | — | — | — | — |
| 5-flucytosine | ≤0.06-16 | 16 | 16 | ≤0.06-16 | 8 | 16 | — | — | — | — |
| Itraconazole ^b^ | 0.12-8 | 0.5 | 1 | 0.03-1 | 0.25 | 1 | — | — | — | — |
| Voriconazole | 0.12-1 | 0.12 | 1 | 0.12-1 | 0.5 | 1 | 87.50 | 0 | 0 | 12.50 |
| Posaconazole ^b^ | 0.03-0.12 | 0.06 | 0.12 | 0.06-0.5 | 0.25 | 0.5 | — | — | — | — |
| Fluconazole ^a^ | 16-128 | 16 | 32 | 16-128 | 64 | 128 | — | — | — | — |
| Anidulafungin | 0.12-0.25 | 0.12 | 0.25 | ≤0.015-0.5 | 0.03 | 0.06 | 93.75 | 0 | 0 | 6.25 |
| Micafungin | 0.06-0.25 | 0.12 | 0.12 | 0.06-0.5 | 0.12 | 0.12 | 93.75 | 0 | 0 | 6.25 |
| Caspofungin | 0.12-0.5 | 0.25 | 0.25 | 0.06-0.5 | 0.25 | 0.25 | 87.50 | 0 | 0 | 12.5 |
| ***Candida parapsilosis*** **(**13) | MIC range | MIC50 | MIC90 | MIC range | MIC50 | MIC90 | CA | VMD | MD | mD |
| Amphotericin B ^b^ | 0.5-1 | 1 | 1 | 0.25-1 | 0.5 | 1 | — | — | — | — |
| 5-flucytosine | ≤0.06-0.25 | ≤0.06 | 0.12 | ≤0.06-0.12 | 0.12 | 0.12 | — | — | — | — |
| Itraconazole ^b^ | 0.06-0.5 | 0.12 | 0.5 | 0.03-0.12 | 0.06 | 0.12 | — | — | — | — |
| Voriconazole | 0.016-1 | 0.03 | 0.5 | ≤0.008-1 | 0.015 | 0.5 | 100 | 0 | 0 | 0 |
| Posaconazole ^b^ | 0.03-0.12 | 0.03 | 0.06 | 0.03-0.12 | 0.06 | 0.12 | — | — | — | — |
| Fluconazole | ≤0.25-32 | 1 | 16 | 0.25-32 | 0.5 | 16 | 84.62 | 0 | 0 | 15.38 |
| Anidulafungin | 0.06-0.5 | 0.25 | 0.5 | 0.015-2 | 1 | 2 | 100 | 0 | 0 | 0 |
| Micafungin | ≤0.03-1 | 1 | 1 | 0.015-2 | 1 | 2 | 100 | 0 | 0 | 0 |
| Caspofungin | 0.12-0.5 | 0.25 | 0.5 | 0.015-1 | 0.5 | 1 | 100 | 0 | 0 | 0 |
| ***Candida tropicalis* (**13) | MIC range | MIC50 | MIC90 | MIC range | MIC50 | MIC90 | CA | VMD | MD | mD |
| Amphotericin B ^b^ | 0.25-1 | 0.5 | 0.5 | 0.5-1 | 1 | 1 | — | — | — | — |
| 5-flucytosine | ≤0.06-0.25 | ≤0.06 | 0.12 | ≤0.06-0.12 | ≤0.06 | ≤0.06 | — | — | — | — |
| Itraconazole ^b^ | 0.12->8 | 0.5 | >8 | 0.12->16 | 0.5 | >16 | — | — | — | — |
| Voriconazole | 0.06->8 | 0.12 | 4 | 0.06->8 | 0.12 | >8 | 92.31 | 0 | 0 | 7.69 |
| Posaconazole ^b^ | 0.03-0.12 | 0.12 | 0.12 | 0.12-1 | 0.25 | 0.5 | — | — | — | — |
| Fluconazole | 1-256 | 2 | 256 | 1->256 | 2 | 256 | 92.31 | 0 | 0 | 7.69 |
| Anidulafungin | 0.06-0.25 | 0.12 | 0.25 | ≤0.015-0.12 | 0.03 | 0.12 | 100 | 0 | 0 | 0 |
| Micafungin | ≤0.03-0.12 | ≤0.03 | 0.06 | 0.015-0.03 | 0.03 | 0.03 | 100 | 0 | 0 | 0 |
| Caspofungin | 0.06-0.25 | 0.12 | 0.25 | 0.015-0.12 | 0.03 | 0.06 | 100 | 0 | 0 | 0 |
| ***Candida lusitaniae* (**13) | MIC range | MIC50 | MIC90 | MIC range | MIC50 | MIC90 | CA | VMD | MD | mD |
| Amphotericin B ^b^ | 0.5-1 | 1 | 1 | ≤0.12-0.25 | 0.25 | 0.25 | — | — | — | — |
| 5-flucytosine | ≤0.06-64 | ≤0.06 | 0.25 | ≤0.06-0.12 | ≤0.06 | ≤0.06 | — | — | — | — |
| Itraconazole ^b^ | 0.06-0.12 | 0.12 | 0.12 | ≤0.015-0.12 | 0.06 | 0.12 | — | — | — | — |
| Voriconazole | ≤0.008-0.03 | 0.016 | 0.03 | ≤0.008-0.015 | 0.015 | 0.015 | — | — | — | — |
| Posaconazole ^b^ | 0.03-0.06 | 0.03 | 0.06 | ≤0.008-0.03 | 0.03 | 0.03 | — | — | — | — |
| Fluconazole ^b^ | ≤0.25-2 | 0.5 | 1 | ≤0.12-1 | 0.5 | 1 | — | — | — | — |
| Anidulafungin ^b^ | 0.12-0.25 | 0.12 | 0.25 | 0.03-0.25 | 0.12 | 0.25 | — | — | — | — |
| Micafungin ^b^ | 0.12-0.25 | 0.12 | 0.25 | 0.015-0.12 | 0.06 | 0.12 | — | — | — | — |
| Caspofungin ^b^ | 0.12-0.25 | 0.25 | 0.25 | 0.015-0.25 | 0.06 | 0.25 | — | — | — | — |
| ***Candida glabrata* (**10) | MIC range | MIC50 | MIC90 | MIC range | MIC50 | MIC90 | CA | VMD | MD | mD |
| Amphotericin B ^b^ | 1-2 | 1 | 2 | 0.25-1 | 0.5 | 1 | — | — | — | — |
| 5-flucytosine | ≤0.06 | ≤0.06 | ≤0.06 | ≤0.06 | ≤0.06 | ≤0.06 | — | — | — | — |
| Itraconazole ^b^ | 0.25-0.5 | 0.5 | 0.5 | 0.25-0.5 | 0.5 | 0.5 | — | — | — | — |
| Voriconazole ^b^ | 0.12-1 | 0.25 | 0.25 | 0.06-0.5 | 0.25 | 0.5 | — | — | — | — |
| Posaconazole ^b^ | 0.12->2 | 0.12 | 1 | 0.25-2 | 0.5 | 1 | — | — | — | — |
| Fluconazole | 4-64 | 16 | 16 | 2-128 | 4 | 16 | 100 | 0 | 0 | 0 |
| Anidulafungin | 0.03-0.25 | 0.06 | 0.12 | ≤0.015-0.12 | 0.03 | 0.06 | 90.00 | 0 | 0 | 10.0 |
| Micafungin | ≤0.03 | ≤0.03 | ≤0.03 | ≤0.008-0.015 | 0.015 | 0.015 | 100 | 0 | 0 | 0 |
| Caspofungin | 0.06-0.5 | 0.12 | 0.25 | 0.03-0.25 | 0.06 | 0.12 | 70.00 | 0 | 0 | 30.0 |
| ***Candida guilliermondii* (**10) | MIC range | MIC50 | MIC90 | MIC range | MIC50 | MIC90 | CA | VMD | MD | mD |
| Amphotericin B ^b^ | 0.5-2 | 1 | 2 | 0.25-1 | 0.25 | 1 | — | — | — | — |
| 5-flucytosine | ≤0.06-0.12 | ≤0.06 | 0.12 | ≤0.06 | ≤0.06 | ≤0.06 | — | — | — | — |
| Itraconazole ^b^ | 0.12-0.5 | 0.25 | 0.5 | 0.12-1 | 0.5 | 1 | — | — | — | — |
| Voriconazole | 0.06-0.5 | 0.12 | 0.12 | 0.03-0.25 | 0.12 | 0.25 | — | — | — | — |
| Posaconazole ^b^ | 0.03-0.25 | 0.12 | 0.25 | 0.06-0.5 | 0.25 | 0.5 | — | — | — | — |
| Fluconazole ^b^ | 2-8 | 4 | 8 | 4-8 | 4 | 8 | — | — | — | — |
| Anidulafungin | 0.25-1 | 0.25 | 0.5 | 0.5-1 | 1 | 1 | 100 | 0 | 0 | 0 |
| Micafungin | ≤0.03-0.5 | 0.25 | 0.5 | 0.25-1 | 0.5 | 1 | 100 | 0 | 0 | 0 |
| Caspofungin | 0.25-0.5 | 0.25 | 0.25 | 0.12-0.5 | 0.25 | 0.5 | 100 | 0 | 0 | 0 |
| ***Cryptococcus neoformans* (**10) | Test range | MIC50 | MIC90 | Test range | MIC50 | MIC90 | CA | VMD | MD | mD |
| Amphotericin B ^b^ | ≤0.03-1 | 0.5 | 1 | 0.5-1 | 0.5 | 1 | — | — | — | — |
| 5-flucytosine^b^ | 2-8 | 4 | 4 | 2-8 | 4 | 8 | — | — | — | — |
| Itraconazole ^b^ | 0.06-0.25 | 0.12 | 0.12 | 0.03-0.12 | 0.06 | 0.12 | — | — | — | — |
| Voriconazole ^b^ | 0.03-0.25 | 0.12 | 0.25 | 0.03-0.12 | 0.06 | 0.12 | — | — | — | — |
| Posaconazole ^b^ | 0.03-0.5 | 0.03 | 0.12 | 0.03-0.12 | 0.06 | 0.12 | — | — | — | — |
| Fluconazole ^b^ | 0.12-16 | 2 | 4 | 0.03-8 | 2 | 8 | — | — | — | — |
| Anidulafungin ^a^ | >16 | >16 | >16 | >8 | >8 | >8 | — | — | — | — |
| Micafungin ^a^ | >32 | >32 | >32 | >8 | >8 | >8 | — | — | — | — |
| Caspofungin ^a^ | 2-16 | 8 | 8 | 2->8 | >8 | >8 | — | — | — | — |
| ***Trichosporon asahii* (**10) | Test range | MIC50 | MIC90 | Test range | MIC50 | MIC90 | CA | VMD | MD | mD |
| Amphotericin B ^b^ | 0.5-1 | 1 | 1 | 0.25-2 | 0.5 | 0.5 | — | — | — | — |
| 5-flucytosine | 2->64 | 4 | 16 | 1->64 | 2 | 64 | — | — | — | — |
| Itraconazole ^b^ | 0.25-0.5 | 0.25 | 0.5 | 0.12-0.5 | 0.25 | 0.25 | — | — | — | — |
| Voriconazole | 0.06-0.25 | 0.06 | 0.12 | 0.06-1 | 0.12 | 0.25 | — | — | — | — |
| Posaconazole ^b^ | 0.06-0.12 | 0.06 | 0.12 | 0.25-0.5 | 0.25 | 0.5 | — | — | — | — |
| Fluconazole ^b^ | 2-16 | 2 | 4 | 4-32 | 8 | 16 | — | — | — | — |
| Anidulafungin ^a^ | >16 | >16 | >16 | >8 | >8 | >8 | — | — | — | — |
| Micafungin ^a^ | >32 | >32 | >32 | >8 | >8 | >8 | — | — | — | — |
| Caspofungin ^a^ | 4 | 4 | 4 | >8 | >8 | >8 | — | — | — | — |
| ***Exophiala dermatitidis*** (22) | Test range | MIC50 | MIC90 | Test range | MIC50 | MIC90 | CA | VMD | MD | mD |
| Amphotericin B | 0.25-8 | 1 | 8 | 0.25-2 | 1 | 2 | — | — | — | — |
| 5-flucytosine | 2->64 | 8 | >64 | 4->64 | >64 | >64 | — | — | — | — |
| Itraconazole | ≤0.016-0.5 | 0.12 | 0.5 | 0.12-0.5 | 0.25 | 0.5 | — | — | — | — |
| Voriconazole | 0.03-0.25 | 0.12 | 0.25 | 0.03-0.25 | 0.12 | 0.25 | — | — | — | — |
| Posaconazole | ≤0.016-1 | 0.06 | 1 | 0.06-0.25 | 0.25 | 0.25 | — | — | — | — |
| Fluconazole | 4-64 | 16 | 64 | 8-64 | 16 | 64 | — | — | — | — |
| Anidulafungin | >16 | >16 | >16 | >8 | >8 | >8 | — | — | — | — |
| Micafungin | >32 | >32 | >32 | >8 | >8 | >8 | — | — | — | — |
| Caspofungin | 4-16 | 8 | 16 | >8 | >8 | >8 | — | — | — | — |

Note: Categorical agreement (CA) was assessed and defined as the percentage of isolates classified in the same category (i.e., susceptible, intermediate, susceptible-dose-dependent, and resistant) between the Droplet 48 (D48) and Sensititre YeastOne (SYO) methods. Very major discrepancy (VMD) was defined as a test result when the D48 result is susceptible and SYO result is resistant. Major discrepancy (MD) was defined as a discrepancy in test results interpreted by D48 as resistant, and the comparator method result was susceptible. Minor discrepancy (mD) was intermediate, and the other was susceptible or resistant. If the MIC value of a method was identified as a range value (e.g., ≤2 or >4), the MIC value of another method that differed from its critical value within ±2 two-fold dilutions or within its range was also considered consistent (e.g., 8 vs. >2, 0.016 vs. ≤2, 2 vs. ≤4; 32 vs. >4).
